# Supplementary material for: Identification of beneficial bacterial strains for tomato growth promotion and biocontrol of bacterial canker caused by Clavibacter michiganensis
Source: Appl Environ Microbiol. 2026 Sep 2;92(9):e01087-26. doi: 10.1128/aem.01087-26 (PMC13599502; doi:10.1128/aem.01087-26)
Supplement: Supplementary file 1 [file aem.01087-26-s0001.pdf]

Experiment 1

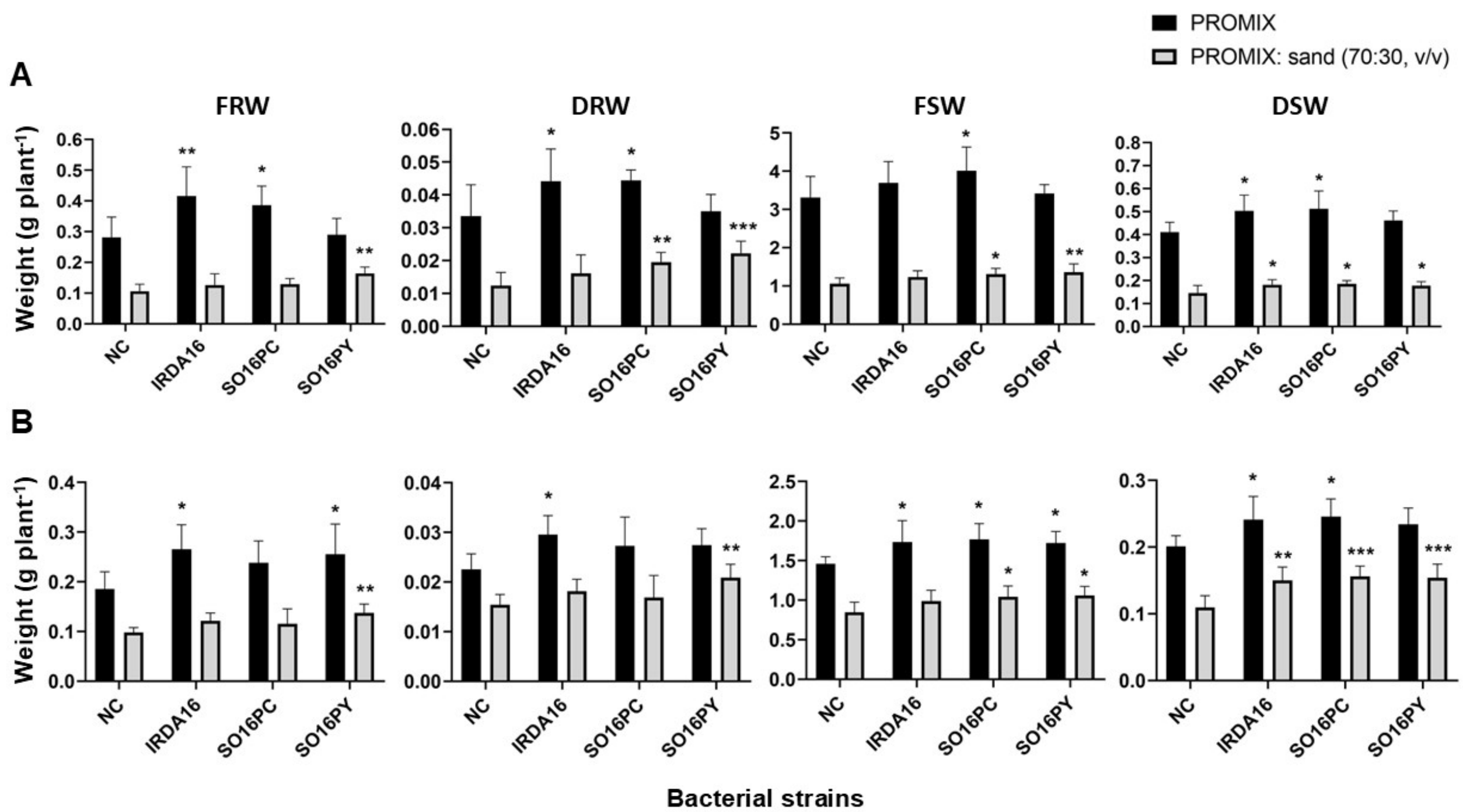

Experiment 2

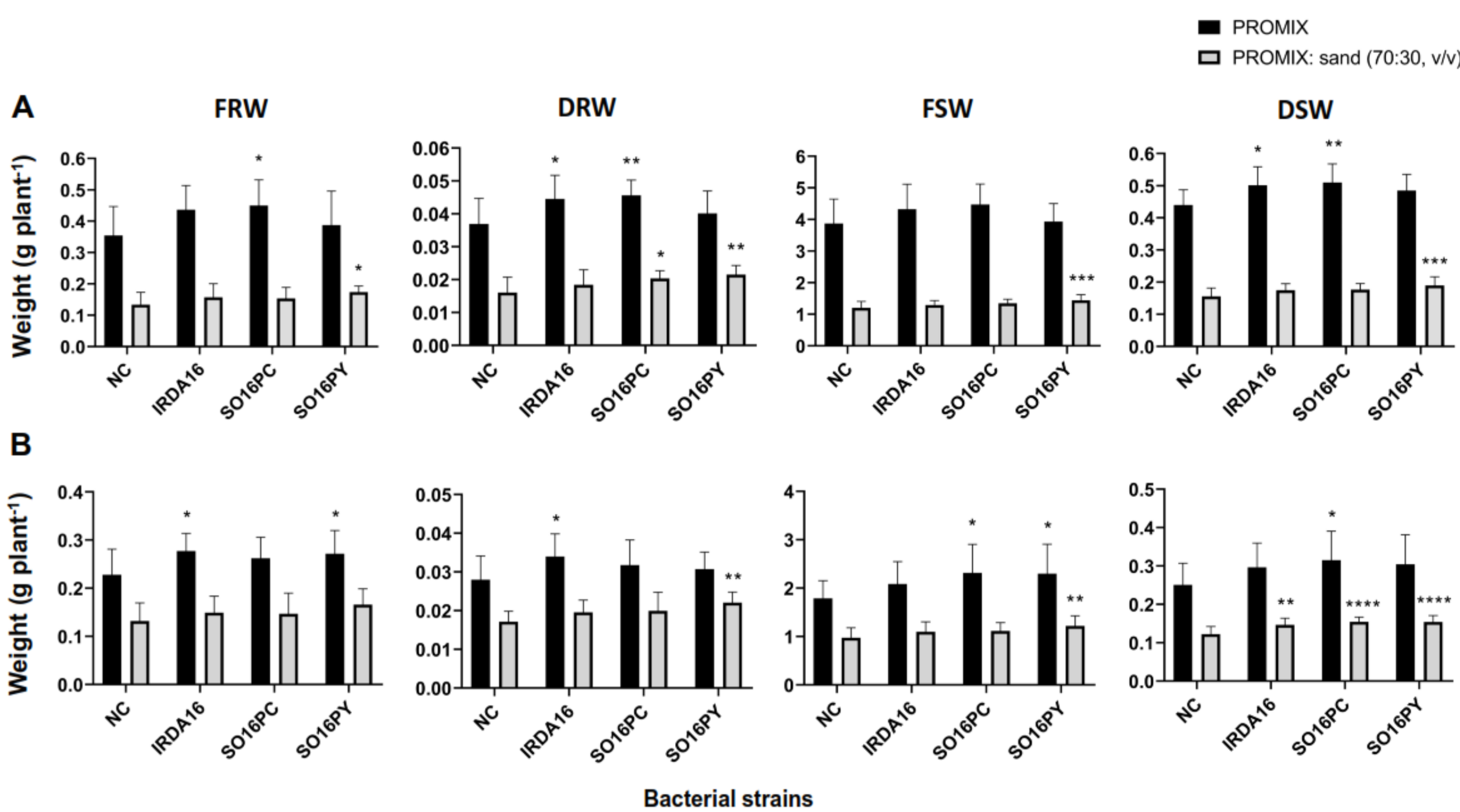

Figure S1. Plant-growth promoting effect of bacterial candidates on tomato growth parameters. Results of the two independent repetitions of the experiment shown in Figure 1. **A.** cultivar Trust, and **B.** cultivar Heinz 2206. Axis X and Y respectively show bacterial strains and plant organs weight per gram. FRW: fresh root weight, DRW: dry root weight, FSW: fresh shoot weight, and DSW: dry shoot weight. Bars indicate means ± standard errors (n=7). NC, negative control (non-inoculated plants treated with sterile distilled water). Asterisks show the significant difference compared to NC: *, $p \leq 0.05$; **, $p \leq 0.01$; and ***, $p \leq 0.001$.

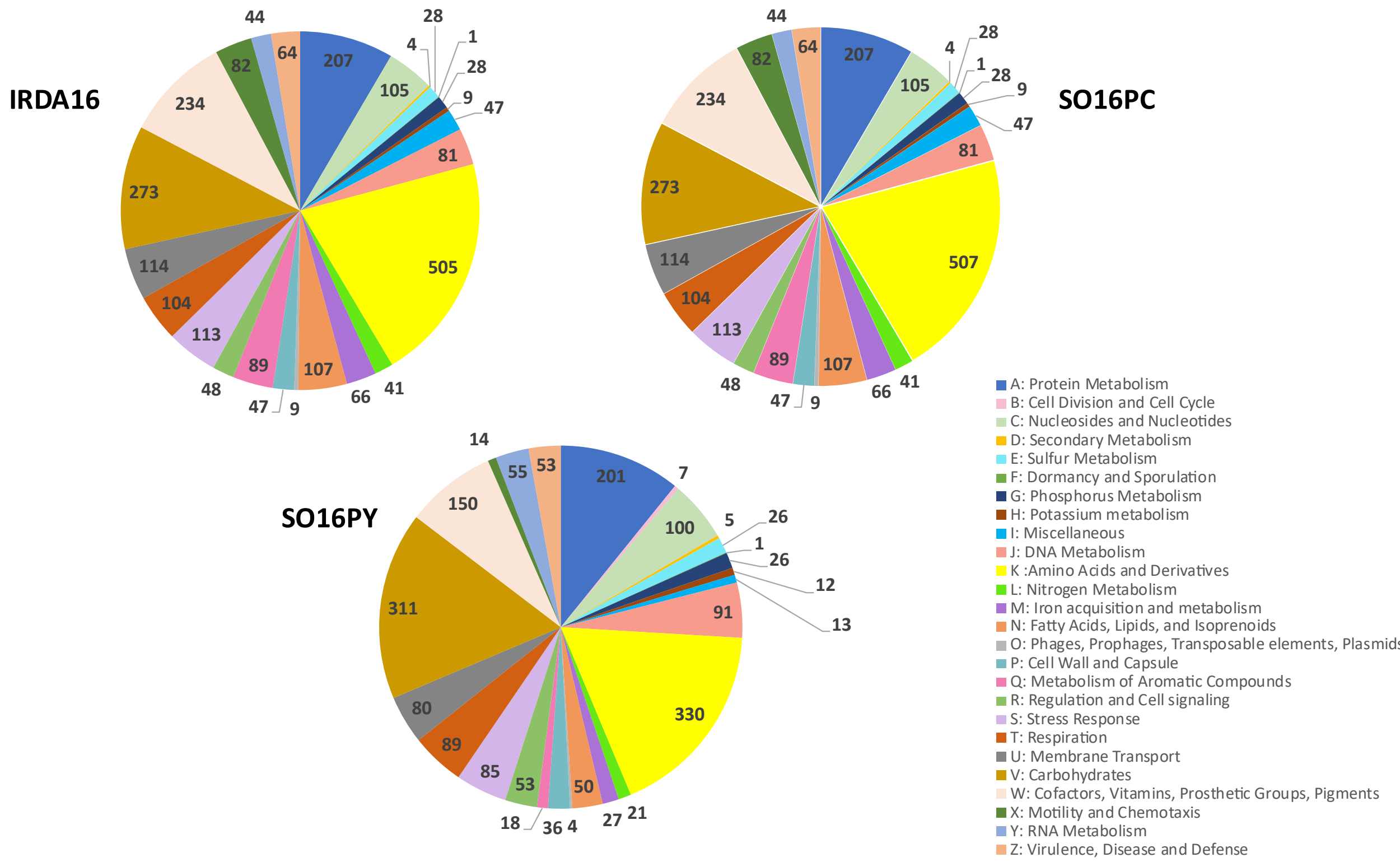

Figure S2. **Clusters of orthologous groups (COG) of the annotated CDSs of bacterial strains.** The SEED-Viewer version 2.0 was used for the identification and prediction of gene clusters in the genomes. Annotated genes were classified into 26 categories. The numbers next to each section indicate the number of genes in each COG cluster.

TABLE S1. *Cm* antagonistic and plant growth-promotion *in vitro* activities of selected strains [a]

Strain [b]	Inhibition Zone [c] (mm^2)	IAA[d] (µg/ml)	Siderophore production[e] (mm^2)	Phosphorus solubilization[f] (mm^2)	Potassium solubilization[g] (mm^2)	ACC [h] deaminase	Reference
Burkholderia vietnamiensis G4 (ED382) [i]	0	3.1±0.3	19.6±0.38	50.2±3.4	56.7±1.5	+	Nelson et al. 1987
Pantoea agglomerants NY60 (ED4177) [j]	683±1.5	21.7±0.5	3.14±0.38	78.5±0.38	19.6±0.38	weak positive	Dagher et al. 2020
Pseudomonas marginalis (7)	132.7±0.38	0	15.9±1.5	23.7±0.38	23.7±3.4	+	Dagher, 2021
Pseudomonas marginalis (IRDA1)	572.5±1.5	0	15.9±0.38	70.8±3.4	28.2±0.38	+	Dagher, 2021
Pseudomonas marginalis (IRDA7)	213.8±3.4	0	9.6±0.38	23.7±1.5	23.7±0.38	+	Dagher, 2021
Pseudomonas poae (FL3F)	491±1.5	0	56.7±3.4	23.7±0.38	4.9±0.38	+	Dagher et al. 2020
Pseudomonas poae (FL4F)	397.6±1.4	0	78.5±0.38	38.4±3.4	19.6±0.38	+	Dagher et al. 2020
Pseudomonas poae (FL9F)	314.1±0.38	0	86.5±6.1	28.2±1.5	23.7±0.38	+	Dagher et al. 2020
Pseudomonas poae FL10F (ED4176)	433.7±1.5	0	103.8±1.5	56.7±1.5	33.1±1.5	+	Dagher et al. 2020
Pseudomonas sp. (41)	363±1.5	0	28.2±0.38	56.7±0.38	38.4±3.4	+	Dagher et al. 2020
Pseudomonas sp. (42)	471.4±6.5	0	38.4±1.5	44.1±6.1	33.1±0.38	+	Dagher et al. 2020
Pantoea sp. (SO16PY)	881.4±0.38	23.7±0.4	4.9±0.38	15.9±0.38	56.7±1.5	+	Lab collection
Pantoea sp. (S63)	615.7±1.5	20.5± 0.3	2.5±0.38	188.7±6.1	50.2±0.38	+	Dagher, 2021
Pseudomonas orientalis (9)	122.7±1.5	0	15.9±0.38	44.1±6.1	15.9±0.38	+	Dagher, 2021
Pseudomonas marginalis (IRDA16)	572.5±6.1	0	33.1±1.5	86.5±1.5	19.6±1.5	+	Dagher, 2021
Pseudomonas donghuensis (M3)	804.2±1.5	12.3±0.2	78.5±1.5	122.7±9.6	28.2±1.5	+	Dagher, 2021
Pseudomonas fluorescens (IRDA4F)	615.7±0.38	0	63.6±1.5	50.2±0.38	23.7±1.5	+	Dagher, 2020
Burkholderia sp. (BC19)	433.7±1.5	0	103.8±3.4	56.7±0.38	50.2±3.4	+	Olishevska et al. 2023
Burkholderia sp. (BC153)	433.7±3.4	0	143.1±6.1	95±1.5	103.8±6.1	+	Olishevska et al. 2023

Continued:

Strain	Inhibition Zone (mm²)	IAA (µg/ml)	Siderophore production (mm²)	Phosphorus solubilization (mm²)	Potassium solubilization (mm²)	ACC deaminase	Reference
Pseudomonas farris (VFB9)	510.7± 6.1	14.3 ± 0.4	28.2 ± 1.5	9.6±0.38	15.9±0.38	+	Olishevska et al. 2023
Burkholderia sola (1702)	213.8±1.5	0	153.9±6.1	113±3.4	78.5±6.1	+	Olishevska et al. 2023
Pseudomonas aeruginosa (416)	593.9±1.5	0	95±1.5	50.2±6.1	19.6±0.38	-	Olishevska et al. 2023
Pseudomonas palmensis (TP19)	226.9±1.5	10 ± 0.2	452.3±6.1	38.4±1.5	28.2±1.5	+	Olishevska et al. 2023
Pseudomonas palmensis (TP48)	103.8±0.38	20.4 ± 0.3	330±1.5	38.4±1.5	33.1±0.38	+	Olishevska et al. 2023
Pseudomonas sp. (TM64)	143.1±6.1	17.7±0.2	33.1±3.4	9.6±0.38	4.9±0.38	+	Olishevska et al. 2023
Pseudomonas farris (TM55)	397.6±3.4	14.5±0.6	15.9±0.38	15.9±0.38	7±0.38	+	Olishevska et al. 2023
Pseudomonas koreensis (To55)	298.6+3.4	0	38.4±1.5	33.1±1.5	4.9±0.38	+	Olishevska et al. 2023
Bacillus pumilus (To79)	471.4±0.38	0	113±6.1	19.6±0.38	3.1±0.38	+	Olishevska et al. 2023
Bacillus sp. (605A)	330±3.4	13.9 ± 0.2	298.6±1.5	33.1±1.5	19.6±0.38	+	Dagher, 2021
Bacillus pumilus (615)	346.3+0.38	0	0	113±1.5	33.1±1.5	+	Dagher, 2021
Erwinia tasmaniensis (9Q)	283.5±1.5	12.6 ±0.2	0	103.8±3.4	103.8±1.5	+	Lab collection
Pantoea agglomerans (NY50)	779.3±3.4	23.7±0.9	2±0.38	86.5±6.1	23.7±0.38	weak positive	Dagher, 2020
Pseudomonas marginalis (SO16PC)	551.5±1.5	0	28.2±0.38	78.5±9.6	23.7±0.38	+	Lab collection

[a] Data are represented as the mean ± standard deviation of two independent replicates.
[b] Strains were identified by 16s rRNA gene sequencing.
[c] Measured area (mm²) for inhibition zone against *Cm* growth.
[d] Indole-3-acetic acid.
[e-g] Measured halo area (mm²).
[h] Aminocyclopropane-1-carboxylate (ACC) deaminase.
[i] Positive control for all PGP tests except IAA test.
[j] Positive control for IAA test.

TABLE S2. Preliminary in planta screening of bacterial strains for tomato (cultivar Trust) growth promotion under growth chamber condition[a].

Treatment	FRW [b]	FSW [c]	DRW [d]	DSW [e]
**Batche 1**				
NC[f]	0.44±0.13	5.02±0.72	0.044±0.019	0.52±0.14
Pantoea agglomerans (NY50)	0.47±0.17	6.02±0.78	0.042±0.019	0.66±0.17
Pseudomonas donghuensis (M3)	0.42±0.09	6.94±0.96**	0.054±0.015	0.79±0.13**
Bacillus pumilus (615)	0.37±0.15	6.67±1.07**	0.044±0.011	0.77±0.12**
Erwinia tasmaniensis (9Q)	0.4±0.15	6.17±0.87	0.045±0.012	0.67±0.09
**Batche 2**				
NC	0.38±0.06	6.6±0.85	0.044±0.013	0.7±0.13
Pseudomonas sp. (42)	0.45±0.05	7.3±1.3	0.042±0.009	0.7±0.18
Pseudomonas fluorescens (IRDA4F)	0.37±0.07	7.3±078	0.037±0.012	0.66±0.19
Pseudomonas sp. (tm64)	0.52±0.12*	7.27±0.7	0.05±0.013	0.82±0.19
**Batche 3**				
NC	0.39±0.12	6.68±0.79	0.067±0.007	0.77±0.11
Pseudomonas poae (FL3F)	0.43±0.1	6.81±0.87	0.062±0.013	0.85±0.17
Pseudomonas poae (FL4F)	0.63±0.14**	7.34±1.1	0.07±0.023	0.81±0.19
Pseudomonas poae (FL9F)	0.56±0.08*	7.34±0.93	0.068±0.015	0.85±0.12
Pseudomonas poae (FL10F)	0.53±0.06	6.9±0.91	0.064±0.013	0.82±0.14
**Batche 4**				
NC	0.29±0.03	5.7±1.04	0.037±0.004	0.56±0.1
Pseudomonas orientalis (9)	0.43±0.05**	6.27±0.91	0.055±0.009**	0.76±0.15*
Pseudomonas marginalis (IRDA16)	0.47±0.1***	5.88±0.83	0.057±0.014**	0.74±0.1*
Pseudomonas sp. (41)	0.23±0.05	3.5±1.3	0.032±0.009	0.42±0.12
Pantoea sp. (SO16PY)	0.43±0.08**	5.41±0.71	0.052±0.009*	0.74±0.11*
**Batche 5**				
NC	0.24±0.03	5.47±0.93	0.034±0.005	0.60±0.17
Pseudomonas palmensis (TP19)	0.28±0.1	5.52±0.98	0.035±0.015	0.57±0.16
Pseudomonas palmensis (TP48)	0.28±0.06	5.35±0.97	0.04±0.01	0.55±0.09
Pseudomonas farris (TM55)	0.34±0.04	6.18±1.2	0.041±0.006	0.64±0.14
Pseudomonas koreensis (TO55)	0.33±0.07	5.8±0.69	0.0414±0.01	0.66±0.13
Pantoea sp. (S63)	0.36±0.15*	5.38±1.16	0.041±0.015	0.51±0.11
**Batche 6**				
NC	0.33±0.1	4.28±1.85	0.042±0.012	0.57±0.21
Bacillus pumilus (TO79)	0.31±0.16	5.04±1.6	0.045±0.022	0.69±0.2
Bacillus sp. (605A)	0.21±0.05	3.85±1.4	0.024±0.005	0.49±0.18
Pseudomonas farris (VFB9)	0.2±0.07	3.77±1.6	0.028±0.008	0.48±0.17
**Batche 7**				
NC	0.37±0.05	4.87±0.41	0.036±0.008	0.43±0.039
Pantoea agglomerans (NY60)	0.36±0.04	4.9±0.71	0.036±0.007	0.43±0.048
Pseudomonas marginalis (IRDA1)	0.4±0.07	5.05±0.57	0.038±0.003	0.43±0.037
Pseudomonas marginalis (IRDA7)	0.37±0.04	4.91±0.33	0.036±0.003	0.42±0.031
Pseudomonas marginalis (7)	0.37±0.03	4.91±0.54	0.035±0.004	0.42±0.042
Pseudomonas marginalis (SO16PC)	0.45±0.04*	5.3±0.34	0.045±0.002*	0.49±0.045*

[a] Data are represented as the mean ± standard deviation of seven replicates.
[b] Fresh root weight
[c] Fresh shoot weight
[d] Dry root weight
[e] Dry shoot weight
[f] Negative control (sterile distilled water)
*, $p \leq 0.05$; **, $p \leq 0.01$; and ***, $p \leq 0.001$.

TABLE S3. Functional annotation of genes associated with plant growth-promoting traits.

**Gene**	**Gene product**	**Function (PGPR trait)**
iucA/iucC	Non-ribosomal peptide synthetase-independent siderophore	Siderophore biosynthesis
NRPS	Non-ribosomal peptide synthetase	Siderophore biosynthesis
pvdS	Alternative sigma factor PvdS	Regulation of Siderophore biosynthesis
pvdP	Pyoverdine biosynthesis protein	Final maturation of Siderophore
tonB	TonB-dependent transporter	Iron-siderophore uptake
fur	Ferric uptake regulator	Iron homeostasis regulation
dfoA	Desferrioxamine biosynthesis protein A	Siderophore biosynthesis
dfoC	Desferrioxamine biosynthesis protein C	Siderophore biosynthesis
fhuA-F	Ferric hydroxamate uptake protein	Iron-siderophore uptake
pqqA–F	PQQ[a] biosynthesis proteins	Phosphate solubilization cofactor
gcd	Glucose dehydrogenase	Phosphate solubilization
kdpA	K+ transporting ATPase subunit A	Potassium uptake
kdpB	K+ transporting ATPase subunit B	Potassium uptake
kdpC	K+ transporting ATPase subunit C	Potassium uptake
acdS	aminocyclopropane-1-carboxylate deaminase	Ethylene regulation
dcyD	D-cysteine desulfhydrase	Stress/ACC[b]-related
iaaM	Tryptophan monooxygenase	IAA[c] biosynthesis (IAM)
iaaH	Indole-3-acetamide hydrolase	IAA biosynthesis (IAM)
ipdC	Indole-3-pyruvate decarboxylase	IAA biosynthesis (IPyA)

[a] Pyrroloquinoline quinone

[b] 1-Aminocyclopropane-1-carboxylic acid

[c] Indole-3-acetic acid

TABLE S4. Identified biosynthetic gene clusters in bacterial strains according to antiSMASH

Region on genome	Biosynthetic gene cluster type	Most similar known cluster from the MIBiG[a] database	Similarity	MIBiG accession number
**Strain IRDA16**				
159881-182106	Terpene	NI[b]	NI	NI
52601-74748	Redox-cofactor	NI	NI	NI
25132-48211	Betalactone	NI	NI	NI
19254-34053	NAGGN[c]	NI	NI	NI
127389-187333	NRPS[d], NRP-metallophore	Viscosin	High	BGC0001312 *Pseudomonas* [*marginalis*] SBW25
30058-59908	NIS[e] Siderophore	NI	NI	NI
1-19136	Terpene	Sodorifen	Medium	BGC0001361 (*Serratia plymuthica*)
3933-33732	Azole-containing-RiPP[f]	Thanamycin	Low	BGC0000443 (*Pseudomonas* sp.)
74418-107123	Arylpolyene	APE Vf[g]	Low	BGC0000837 (*Aliivibrio fischeri*)
54649-83809	NRPS	Pyoverdine SXM-1	Low	BGC0002693 (*Pseudomonas* sp.)
1-5586	NRPS	Crochelin A	Low	BGC0002001 (*Azotobacter chroococcum*)
1-2453	NRPS	Nunapeptin, nunamycin	Low	BGC0001416 (*Pseudomonas* sp.)
1-3824	NRPS	NI	NI	NI
72921-114881	NRPS	NI	NI	NI
59629-125636	NRPS, NRP-metallophore	NI	NI	NI
62607-99921	NRPS	NI	NI	NI
**Strain SO16PC**				
242705-264930	Terpene	NI	NI	NI
224278-246425	Redox-cofactor	NI	NI	NI
125184-148263	Betalactone	NI	NI	NI
80829-95628	NAGGN	NI	NI	NI
30058-59908	NIS Siderophore	NI	NI	NI
3111-15931	Hydrogen cyanide	NI	NI	NI
127582-187526	NRPS, NRP-metallophore	Viscosin	High	BGC0001312 *Pseudomonas* [*marginalis*] SBW25

Continued:

Region on the Genome	Biosynthetic gene cluster type	Most similar known cluster from the MIBiG database	Similarity	MIBiG accession number
154259-173394	Terpene	Sodorifen	Medium	BGC0001361 (*Serratia plymuthica*)
3933-33732	Azole-containing-RiPP	Thanamycin	Low	BGC0000443 (*Pseudomonas* sp.)
74418-107126	Arylpolyene	APE Vf	Low	BGC0000837 (*Aliivibrio fischeri*)
1-29116	NRPS	Pyoverdine SXM-1	Low	BGC0002693 (*Pseudomonas* sp.)
1-5586	NRPS	Crochelin A	Low	BGC0002001 (*Azotobacter chroococcum*)
1-2453	NRPS	Nunapeptin, nunamycin	Low	BGC0001416 (*Pseudomonas* sp.)
1-3824	NRPS	NI	NI	NI
59629-125636	NRPS, NRP-metallophore	NI	NI	NI
62607-99921	NRPS	NI	NI	NI
**Strain SO16PY**				
92062-114228	Redox-cofactor	NI	NI	NI
140010-154646	hserlactone	NI	NI	NI
47987-74243	Azole-containing-RiPP	NI	NI	NI
13564-43918	Siderophore	Desferrioxamine E	High	BGC0001572 (*Pantoea agglomerans*)
41469-67233	Terpene	Carotenoid	High	BGC0000638 (*Pantoea ananatis*)
1-46206	NRP-metallophore, NRPS	Frederiksenibactin	High	BGC0002413 (*Yersinia frederiksenii*)
60976-102773	Arylpolyene	Aryl polyenes	High	BGC0002008 (*Xenorhabdus doucetiae*)
106524-127000	Phenazine	Phenazine	Medium	BGC0001868 (*Xenorhabdus szentirmaii*)

[a] Minimum Information about a Biosynthetic Gene cluster.
[b] NI: Not identified.
[c] NAGGN: N-acetylglutaminylglutamine amide.
[d] NRPS: Non-ribosomal peptides synthetases.
[e] NRPS-independent siderophores.
[f] RiPP: Ribosomally synthesized and post-translationally modified peptides.
[g] APE Vf: arylpolyene.

TABLE S5. Identified biosynthetic gene clusters in reference strain of *P. marginalis* DSM 13124^{T} to antiSMASH

Biosynthetic gene cluster type	From	To	Similarity confidence	Most similar known cluster from the MIBiG[a] database	MIBiG accession number
NRPS[b]	80,387	130,684	Low	Pyoverdine SXM-1	BGC0002693 *(Pseudomonas* sp.*)*
Redox-cofactor	399,414	421,561	NI[c]	NI	NI
arylpolyene	84,292	127,867	Low	APE-vf[d]	BGC0000837 (*Aliivibrio fischeri* ES114)
Siderophore	6,624	36,474	NI	NI	NI
betalactone	34,751	50,873	NI	NI	NI
NRPS	285,703	338,599	NI	NI	NI
NAGGN[e]	371,788	386,595	NI	NI	NI
NRP-metallophore	1	28,344	NI	NI	NI
Hydrogen-cyanide	1	12,162	NI	NI	NI
butyrolactone	25,584	36,594	NI	NI	NI

[a] Minimum Information about a Biosynthetic Gene cluster.
[b] NRPS: Non-ribosomal peptides synthetases.
[c] Not identified.
[d] APE Vf: arylpolyene.
[e] NAGGN: N-acetylglutaminylglutamine amide.

TABLE S6. Bacterial strains used in phylogenetic study.

Type Strain	Accession number
***Pseudomonas* spp.**	
Pseudomonas marginalis ICMP 3553	PRJNA292453
Pseudomonas marginalis DSM 13124	PRJNA548434
Pseudomonas marginalis PS92	PRJNA956831
Pseudomonas marginalis MGMM3	PRJNA960949
Pseudomonas marginalis SBW25	PRJNA1047486
Pseudomonas marginalis M13	PRJNA1183984
Pseudomonas marginalis DSM 18529	PRJNA548434
Pseudomonas marginalis PgKB35	PRJNA560751
Pseudomonas marginalis IT-357P	PRJEB59762
Pseudomonas marginalis ORh26	PRJNA1194393
Pseudomonas marginalis WS 4668	PRJNA612806
Pseudomonas marginalis Qb1D1	PRJNA1193087
Pseudomonas marginalis Qb1D2	PRJNA1193088
Pseudomonas marginalis EDr2-2	PRJNA1165332
Pseudomonas marginalis EDr2-4	PRJNA1165332
Pseudomonas marginalis SR17	PRJNA844386
Pseudomonas marginalis Phyllo_191	PRJNA961062
Pseudomonas marginalis FSL S12-0184	PRJNA1003870
Pseudomonas marginalis FSL S12-0074	PRJNA1003870
Pseudomonas marginalis H21	PRJNA276809
Pseudomonas marginalis H14	PRJNA276807
Pseudomonas marginalis PA-2-2B	PRJNA587608
Pseudomonas allii MAFF 131514	PRJNA635745
Pseudomonas antarctica DSM 151318	PRJEB29955
Pseudomonas asgharzadehiana SWRI132	PRJNA639797
Pseudomonas aylmerensis S1E40	PRJNA327239
Pseudomonas azadiae SWRI103	PRJNA639797
Pseudomonas azotoformans DSM 18862	PRJNA350775
Pseudomonas canadensis Feb-92	PRJNA224560
Pseudomonas cedrina DSM 17516	RJNA350775
Pseudomonas costantinii LMG 22119	PRJNA311471
Pseudomonas cremoris WS 5106	PRJNA622431
Pseudomonas cyclaminis MAFF 301449	PRJNA670593
Pseudomonas edaphica RD25	PRJNA543005
Pseudomonas extremaustralis DSM 17835	PRJEB15798
Pseudomonas extremorientalis DSM15824	PRJEB29960

Continued:

**Type Strain**	**Accession number**
Pseudomonas fildesensis KG01	PRJNA287706
Pseudomonas grimontii DSM 1515	PRJNA303779
Pseudomonas kairouanensis KC12	PRJNA485038
Pseudomonas karstica CCM 7891	PRJNA587868
Pseudomonas kitaguniensis MAFF 212408	PRJNA563800
Pseudomonas lactucae MAFF 301380	PRJNA702606
Pseudomonas libanensis DSM 17149	PRJNA274345
Pseudomonas lurida LMG 21995	PRJNA303504
Pseudomonas nabeulensis E10B	PRJNA485041
Pseudomonas orientalis DSM 17489	PRJNA274345
Pseudomonas palleroniana LMG 23076	PRJNA445872
Pseudomonas petroselini MAFF 311094	PRJNA785388
Pseudomonas pisciculturae P115	PRJNA662597
Pseudomonas poae LMG 21465	PRJNA338673
Pseudomonas putida NBRC 14164	PRJDB191
Pseudomonas rhodesiae CIP104664	PRJNA709559
Pseudomonas salomonii LMG 22120	PRJNA338689
Pseudomonas simiae CCUG 50988	PRJEB17091
Pseudomonas sivasensis P7	PRJNA611068
Pseudomonas spelaei CCM 7893	PRJNA587868
Pseudomonas synxantha LMG 2190	PRJNA323046
Pseudomonas tolaasii DSM 19342	PRJEB29963
Pseudomonas tritici SWRI145	PRJNA639797
Pseudomonas trivialis LMG 21464	PRJNA338684
Pseudomonas veronii DSM 11331	PRJNA274345
Pseudomonas yamanorum LMG 27247	PRJEB16453
***Pantoea* spp.**	
Pantoea agglomerans LMG 1286	PRJNA231221
Pantoea alhagi LTYR-11Z	PRJNA374633
Pantoea allii LMG 24248	PRJNA403848
Pantoea ananatis LMG 2665	PRJNA240762
Pantoea anthophila LMG 2558	PRJNA549748
Pantoea brenneri LMG 5343	PRJNA252984
Pantoea conspicua LMG 24534	PRJNA252986
Pantoea cypripedii LMG 2657	PRJNA252995
Pantoea deleyi LMG 24200	PRJNA705825

Continued:

Type Strain	Accession number
Pantoea dispersa CCUG 25232	PRJNA563568
Pantoea endophytica 596	PRJNA422728
Pantoea eucalypti LMG 24197	PRJNA503936
Pantoea eucrina LMG 5346	PRJNA252988
Pantoea gaviniae DSM 22758	PRJNA430362
Pantoea intestinalis SRCM103226	PRJNA438180
Pantoea latae AS1	PRJNA376797
Pantoea rodasii LMG 26273	PRJNA252990
Pantoea rwandensis LMG 26275	PRJNA252996
Pantoea septica LMG 5345	PRJNA252991
Pantoea stewartii CCUG 26359	PRJNA563568
Pantoea theicola QC88-366	PRJNA407784
Pantoea vagans LMG 24199	PRJNA505269
Pantoea wallisii LMG 26277	PRJNA252993
***Brenneria* sp.**	
Brenneria alni NCPPB 3934	PRJNA342025

References

Dagher, F., Nickzad, A., Zheng, J., Hoffmann, M., and Déziel, E. 2021. Characterization of the biocontrol activity of three bacterial isolates against the phytopathogen *Erwinia amylovora*. MicrobiologyOpen. 10: e1202.

Dagher, F., Olishevska, S., Philion, V., Zheng, J., and Déziel, E. 2020. Development of a novel biological control agent targeting the phytopathogen *Erwinia amylovora*. Heliyon. 6: e05222.

Nelson, M. J., Montgomery, S. O., Mahaffey, W. R., and Pritchard, P. 1987. Biodegradation of trichloroethylene and involvement of an aromatic biodegradative pathway. Appl. Environ. Microbiol. 53: 949-954.

Olishevska, S., Nickzad, A., Restieri, C., Dagher, F., Luo, Y., Zheng, J., and Déziel, E. 2023. *Bacillus velezensis* and *Paenibacillus peoriae* strains effective as biocontrol agents against *Xanthomonas* bacterial spot. Appl. Microbiol. 3: 1101-1119.
